# Association Between Fatigue and Cognitive Impairment at 6 Months in Patients With Ischemic Stroke Treated With Acute Revascularization Therapy

**DOI:** 10.3389/fneur.2019.00931

**Published:** 2019-08-28

**Authors:** Mathilde Graber, Lucie Garnier, Gauthier Duloquin, Sophie Mohr, Sophie Guillemin, Océane Ramaget, Ariane Piver, Cécile Tainturier, Christine Bret-Legrand, Benoit Delpont, Christelle Blanc-Labarre, Julien Guéniat, Marie Hervieu-Bègue, Guy-Victor Osseby, Maurice Giroud, Yannick Béjot

**Affiliations:** Dijon Stroke Registry, EA7460, Pathophysiology and Epidemiology of Cerebro-Cardiovascular diseases (PEC2), University Hospital of Dijon, University of Burgundy, Dijon, France

**Keywords:** stroke, ischemic stroke, fatigue, cognitive impairment, outcome, depression

## Abstract

**Background:** Fatigue is a frequent symptom after stroke. We aimed to determine the association between fatigue and cognitive performance in patients with ischemic stroke who received acute revascularization therapy (IV thrombolysis and/or mechanical thrombectomy).

**Methods:** Seventy patients were prospectively included in the stroke unit of the University Hospital of Dijon, France. A follow-up was performed at 6 months with clinical examination, fatigue assessment by the Fatigue Severity Scale (FSS), and a comprehensive neuropsychological evaluation. Patients with fatigue (FSS score >4) were compared with patients without fatigue. Neuropsychological factors associated with fatigue at 6 months were analyzed using multivariable logistic regression models.

**Results:** Fatigue was reported by 34.3% of patients. Patients with fatigue were older, had more frequent residual handicap, depressive symptoms, and impaired quality of life. They had more frequently low score (<26) on the MoCA scale (79.2 vs. 47.8%, OR = 4.15; 95% CI: 1.32–13, *p* = 0.015), memory impairment (60 vs. 30.6%, OR = 3.41; 95% CI: 1.09–10.7, *p* = 0.035), and executive dysfunction (65 vs. 30.8%, OR = 4.18; 95% CI: 1.33–13.1, *p* = 0.014). In multivariable logistic regression analysis, only memory impairment was independently associated with fatigue (OR = 5.70; 95% CI: 1.09–29.6, *p* = 0.039). Further analyses restricted to non-depressed patients (*n* = 58, 84.1%) showed in multivariable models that a score < 26 on MoCA scale (OR 5.12; 95% CI: 1.00–26.2, *p* = 0.05), and a memory impairment (OR = 6.17; 95% CI: 1.06–35.9, *p* = 0.043) were associated with fatigue. There was also a non-significant trend toward an association between divided attention deficit and fatigue (OR = 6.79; 95% CI: 0.80–57.6, *p* = 0.079).

**Conclusion:** The association between fatigue and subtle cognitive impairment including memory or attention deficits could be of interest in elaborating future interventional studies to evaluate the impact of therapeutic strategies, including cognitive rehabilitation, on fatigue.

## Introduction

Ischemic stroke has beneficiated from major therapeutic advances over the past 20 years, leading to improvements in patients' survival and motor outcome. Consequently, several previously neglected post-stroke complications have emerged as important concerns affecting quality of life. Fatigue is a major complaint of patients after ischemic stroke. Fatigue is a subjective, multidimensional experience, with perceptual-motor, emotional and cognitive components, making it difficult to define and measure. Objective fatigue, defined as an observable and measurable decrease in performance that occurs during the repetition of a mental or physical activity, can be distinguished from subjective fatigue, defined as a feeling of exhaustion, weakness, and aversion to effort (1). For Staub and Bogousslavsky, it is a reversible decrease or loss of ability, associated with an intense feeling of physical and/or mental overwork, even in the absence of special effort, and a pronounced feeling of exhaustion that disrupts or makes it impossible to perform routine activities (2).

The prevalence of fatigue after ischemic stroke ranges between 29 and 77% in the literature (3). Several reasons can explain these inconsistent estimates such as the use of various scales for assessment, various time points after stroke for evaluation, differences in population settings, and characteristics of patients including residual motor disability, chronic pain, sleep disorders, and depression, that may influence post-stroke fatigue (4). Fatigue can persist long after the onset of the cerebral infarction and has a negative impact in terms of survival, recovery, and be associated with a profound deterioration in several aspects of daily life (family, social, and professional) (5).

Although it is the subject of a growing literature, the exact mechanisms underlying post-stroke fatigue have not yet been fully elucidated. Some pathophysiological studies carried out on various neurological diseases suggest that primary fatigue could be linked to damage in neural networks, particularly reticular formation and attention network (6–9). Surprisingly, while cognitive impairment is a major complication after stroke (10), there is few data on its association with fatigue. Indeed, a very limited number of studies focusing on post-stroke fatigue incorporated cognitive assessment of patients, and assessments were most often based on global tests or limited to a few areas of cognition, thus making conclusions uncertain (11).

Therefore, the aim of this study was to investigate the prevalence of fatigue 6 months after ischemic stroke and its association with cognitive performances in patients who received acute revascularization therapy.

## Methods

### Study Population

This observational prospective cohort study was conducted at the Stroke Unit of the University Hospital of Dijon, France (Study Registration Number 2017-A01906-47; Clinical Trial Number NCT03288090). This study received approval from the national ethics committee (CPP SUD-EST 4). All patients gave their oral consent to participate according to the French legislation.

Patients ≥18 years old who suffered hemispheric acute ischemic stroke defined according to the WHO criteria and confirmed by imaging (CT-scan or MRI) who received a revascularization therapy (intravenous thrombolysis and/or mechanical thrombectomy) according to current recommendations were included between August 2017 and May 2018. Exclusions criteria were major co-morbidities prior to ischemic stroke that may be responsible for significant fatigue, previous dementia or aphasia, persistence of severe aphasia, or severe disability (modified Rankin scale score > 3) at 6 months preventing a reliable cognitive assessment. Because of the exploratory nature of this study in the absence of previous reliable data in ischemic stroke patients treated with acute revascularization therapy, no sample size calculation was performed.

### Data Collected, Patients' Follow-Up, and Outcomes Measured

At inclusion following data were collected: demographics, vascular risk factors including hypertension (high blood pressure recorded in a patient's medical history or patients under antihypertensive treatment), diabetes mellitus (reported in the medical record or patients taking insulin or oral hypoglycemic agents), hypercholesterolemia (reported in the medical history or patients treated with lipid-lowering therapy), current smoking, history of coronary heart disease, atrial fibrillation, excessive alcohol consumption (defined as alcohol intake ≥3 units a day in men and ≥2 in women). Stroke severity at onset was evaluated using the National Institutes of Health Stroke Scale (NIHSS) score.

Patients were evaluated face-to-face by a senior neurologist at 6 months. Clinical examination included evaluation of the NIHSS score, and functional handicap using the modified Rankin Score (mRS). Fatigue was evaluated by the Fatigue Severity Scale (FSS) (12). This scale includes 9 items rated from 1 to 7, assessing physical fatigue, the impact of fatigue on the psychosocial environment and fatigue in general (3 items in each category). Each item is a quote for which patients are asked to judge the application for themselves of “strongly disagree” (1 point) to “strongly agree” (7 points). The total score therefore varies from 9 to 63. The average of the total score is calculated by dividing the total score by the number of items to which the patient has responded. This average defines the FSS score, which ranges from 1 to 7. Patients with a score >4 were considered as having fatigue in accordance with the literature (13). A vertical visual numerical scale for fatigue assessment was also performed in addition to the FSS scale (rated from 0 to 100 on a 10 cm scale). Anxiety and depression were rated using the Hospital Anxiety Depression scale (HAD). A score >7 for either anxiety or depression was retained as significant (14). Global pain and quality of life were rated on a vertical numerical scale (rated from 0 to 100 on a 10 cm scale). A sleep assessment was also performed using the Pittsburgh Sleep Quality Index (PSQI). A total score >5 was considered to reflect sleep disturbances.

A comprehensive neuropsychological assessment was performed by a neuropsychologist according to the GRECoGVASC battery (15, 16), a French-language adaptation and standardization of the National Institute of Neurological Disorders and Stroke–Canadian Stroke Network comprehensive battery (17). Several cognitive domains were explored: visuospatial and visuoconstructive skills assessed by the Rey Figure, episodic memory (verbal and visual) evaluated by the RL/RI-16 test [French adaptation of the Free and Cued Selective Reminding Test of Gruber and Buschke (18)] for the verbal modality and memory reproduction of the Rey figure for the visual modality (19), executive functions evaluated by the Trail Making Test (TMT) (20), the Subtest of Codes from Wechsler adult intelligence scale (WAIS-IV) (21), and the Verbal Fluences (categorical and lexical) (22). To assess divided attention, the computerized double task divided attention test of the Test of Attentional Performance (TAP) was administered to patients (23). Working memory was also tested using the Corsi cubes test and the serial span test taken from the Wechsler Memory Scale (MEM III) (24). Finally, global cognitive function was evaluating by the Montreal Cognitive Assessment scale (MoCA) (25).

The results of the neuropsychological evaluation were dichotomized into two groups (normal or abnormal). The standards chosen were defined according to the GREFEX (French Focus Group on Executive Functions Assessment) standards for the evaluation of executive functions (22), and the GREMEM (French Focus Group on Memory Assessment) standards for the evaluation of memory functions (26). These norms are stratified on age and socio-cultural level. Concerning the interpretation of the tests from the MEM III and WAIS-IV scales, the pathological thresholds were defined on the basis of standard scores, stratified on age. Working memory and/or information processing speed were considered abnormal if a standard score was ≤ 5 for one of the scores in each event. The pathological threshold for the divided attention test was ≤ 10th percentile for the number of omissions, as used in clinical practice. Patients were considered to have executive functions impairment if they had an abnormal test in at least one of the following domains: mental flexibility, fluences, working memory, or processing speed.

### Statistical Analysis

Categorical variables were presented as frequencies, and quantitative variables were summarized as mean ± standard deviation (SD), or median (interquartile interval, IQR) values. Two groups of patients were considered: patients who had significant fatigue at 6 months (FSS scale >4) vs. patients without fatigue. Characteristics of patients were compared between groups using the Chi-2 test or exact Fisher test for categorical variables, and the Wilcoxon- Mann-Whitney test for quantitative variables. Logistic regression models were used to evaluate factors associated with fatigue at 6 months. In multivariate analyses, confounding variables with a *p* < 0.10 were introduced into the final models. A sub-group analysis was performed in non-depressed patients. Statistical analysis was performed with STATA@13 software (StataCorp LP, College Station, Texas, USA).

## Results

### Prevalence of Fatigue and Patients' Characteristics

**Figure 1:**
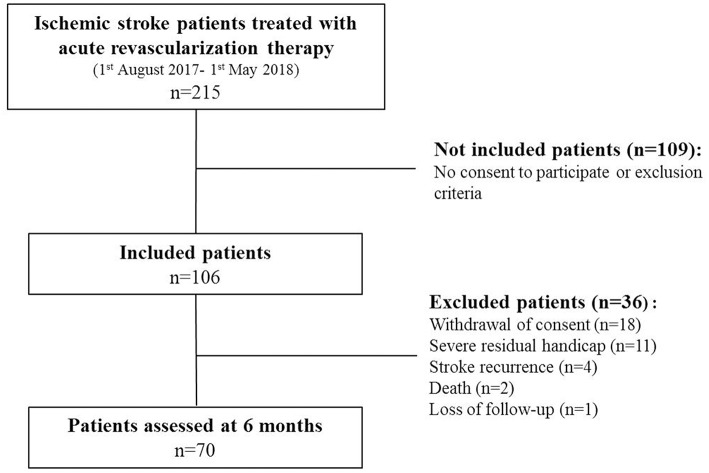
Flowchart of the study.

Of the 215 patients admitted for acute ischemic stroke who received a revascularization therapy between August 2017 and May 2018, 106 were initially included. Among these patients, 70 were finally assessed at 6 months (59% men, mean age: 65.7 ± 14.5 years). Reasons for exclusion of patients were withdrawal of consent during follow-up (*n* = 18), severe residual handicap not compatible with a neuropsychological evaluation (*n* = 11), stroke recurrence (*n* = 4), death (*n* = 2), or loss of follow-up (*n* = 1) (Figure 1).

All patients responded to all items of the FSS. There was a good correlation between FSS score and score on fatigue vertical numerical scale (Spearman coefficient: 0.66, *p* < 0.001). Twenty-four patients (34.3%) reported significant fatigue (defined as FSS score >4). Characteristics of patients according to fatigue are shown in Table 1. Patients who reported fatigue were older (70.3 ± 13.1 vs. 63.3 ± 14.7 *p* = 0.04), and had more frequently hypercholesterolemia, depressive symptoms (29.2 vs. 8.9%, *p* = 0.028), and residual handicap (mRS score ≥2: 58.3% vs. 28.3, *p* = 0.014). In addition, patients with fatigue had worse quality of life on the vertical numerical scale [Median score: 50 (47–67) vs. 75 (65–80), *p* = 0.0005]. In multivariable analysis, factors associated with significant fatigue at 6 months were a history of hypercholesterolemia, the presence of depressive symptoms on the HAD scale, mRS score ≥2 and, lower score on quality of life scale (Table 2).

**Table 1:** Characteristics of patients according to fatigue at 6 months.

	**No fatigue**		**Fatigue**		***P***
	***N*** **=** **46 (65.7%)**		***N*** **=** **24 (34.3%)**		
	***N***	**%**	***N***	**%**	
Age, mean ± SD	63.3 ± 14.7		70.3± 13.1		0.04
Age, median (IQR)	63 (54–73)		75 (64–78)		
Male gender	30	65.2	11	45.8	0.12
**Medical history**					
Diabetes	7	15.2	3	12.5	0.53
Hypertension	24	52.2	15	62.5	0.41
Hypercholesterolemia	12	26.1	12	50.0	0.045
Coronary heart disease	6	13.0	1	4.2	0.23
Atrial fibrillation^$^	10	22.2	6	25.0	0.80
Current smoker	14	30.4	5	20.8	0.39
Alcohol consumption	8	17.4	2	8.3	0.26
Premorbid mRS score <2	43	93.5	23	95.8	0.58
• NIHSS score at onset, median (IQR)	8 (5–14)		10 (5–15)		0.69
**Acute revascularization therapy**					0.30
IV thrombolysis only	26	56.5	10	41.7	
• Mechanical thrombectomy only	8	17.4	8	33.3	
Combined therapy	12	26.1	6	25.0	
**Stroke side**					0.32
Right	18	39.1	13	54.2	
Left	25	54.4	11	45.8	
Bilateral	3	6.5	0	0	
**Outcomes at 6 months**					
mRS score					0.014
*mRS 0–1*	33	71.7	10	41.7	
*mRS ≥ 2*	13	28.3	14	58.3	
NIHSS score = 0	37	80.4	14	58.3	0.048
Anxiety	12	26.7	8	33.3	0.56
Depressive symptoms	4	8.9	7	29.2	0.028
• Depressive symptoms or antidepressant	8	17.8	10	41.7	0.031
Sleep disturbances	19	43.2	16	66.7	0.06
Pain, mean ± SD^†^	22.8 ± 22.1		33.3 ± 24.5		0.07
Pain, median (IQR)^†^	20 (0–35)		40 (7–50)		
Quality of life, mean ± SD^†^	72.33 ± 15.43		56.67 ± 19.87		<0.01
Quality of life, median (IQR)^†^	75 (65–80)		50 (47.5–67.5)		

$ *Previously known or diagnosed during hospitalization*.

† *Numerical vertical scale*.

**Table 2:** Factors independently associated with fatigue at 6 months in multivariable logistic regression analysis.

	**OR (95% CI)**	***P***
Hypercholesterolemia	2.83 (1.0–7.98)	0.049
mRS score ≥ 2	3.55 (1.26–10.0)	0.016
Depressive symptoms	4.22 (1.09–16.3)	0.037
Quality of life^†^	0.95 (0.92–0.98)	0.002

*OR, Odd ratios*.

† *Numerical vertical scale*.

### Neuropsychological Evaluation and Fatigue at 6 Months

**Figure 2:**
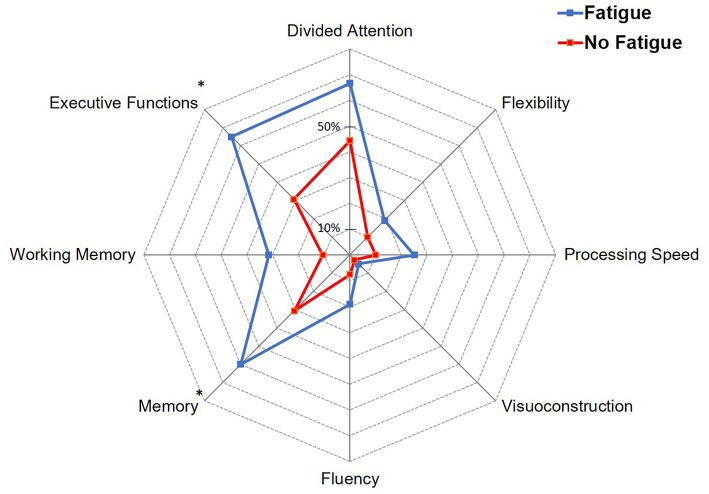
Proportion of patients with impairment in the different cognitive domains according to fatigue. *p < 0.05.

There was no difference in the proportion of patients who were administered neuropsychological tests according to fatigue at 6 months (Table 3). A higher proportion of patients with a low score on the MoCA scale (score <26) was observed in patients with fatigue compared with patients without fatigue (79.2 vs. 47.8%, OR = 4.15; 95% CI: 1.32–13, *p* = 0.015). Figure 2 shows the proportion of patients with abnormal scores on the different cognitive domains according to fatigue. A higher proportion of patients with fatigue had memory impairment (60 vs. 30.6%, OR = 3.41; 95% CI: 1.09–10.7, *p* = 0.035), and executive dysfunction defined as impairment of either working memory, processing speed, fluency or mental flexibility (65 vs. 30.8%, OR = 4.18; 95% CI: 1.33–13.1, *p* = 0.014). In multivariable logistic regression analysis, memory impairment was the only cognitive variable independently associated with fatigue at 6 months (OR = 5.70; 95% CI: 1.09–29.6, *p* = 0.039; Table 4).

**Table 3:** Proportion of patients who performed neuropsychological tests in fatigue and no fatigue groups.

	**No fatigue**		**Fatigue**		***P***
	***N*** **=** **46 (65.7%)**		***N*** **=** **24 (34.3%)**		
	***N***	**%**	***N***	**%**	
RL/RI-16	39	84.8	21	87.5	0.76
Rey figure copy	39	84.8	21	87.5	0.76
Rey figure memory reproduction	36	78.3	18	75.0	0.76
TMT	41	89.1	21	87.5	0.84
Subtest of Codes (WAIS-IV)	40	87.0	20	83.3	0.68
Verbal Fluences	40	87.0	21	87.5	0.95
Working memory (MEM III)	38	82.6	19	79.2	0.73
TAP	36	78.3	15	62.5	0.16
MOCA	46	100	24	100	1.00

**Table 4:** Association between fatigue at 6 months and each neuropsychological variable in overall patients and non-depressed patients in multivariable logistic regression analyses.

	**Tests used**	**Overall patients**		**Non-depressed patients**	
		**OR (95% CI)^**§**^**	**P**	OR (95% CI)^*^	**P**
MoCA <26	–	2.52 (0.63–10.0)	0.189	5.12 (1.00–26.2)	0.05
Memory impairment	RL/RI-16 Rey figure memory reproduction	5.70 (1.09–29.6)	0.039	6.17 (1.06–35.9)	0.043
Divided attention impairment	TAP	3.05 (0.59–15.8)	0.185	6.79 (0.80–57.6)	0.079
Working memory impairment	Subtest of Codes (WAIS-IV) Working memory (MEM III)	2.69 (0.44–16.4)	0.283	2.27 (0.35–14.7)	0.388
Processing speed impairment	TMT part A Subtest of Codes (WAIS-IV)	2.16 (0.33–13.9)	0.419	1.77 (0.25–12.6)	0.567
Mental flexibility impairment	TMT part B	1.83 (0.32–10.6)	0.501	3.24 (0.39–26.8)	0.275
Fluences impairment	Verbal Fluences	1.11 (0.13–9.52)	0.925	1.49 (0.15–14.9)	0.735
Executive functions impairment^†^		2.01 (0.5–8.06)	0.327	2.59 (0.54–12.4)	0.234

*OR, odd ratios*.

§ *Adjusted for age, sex, mRS score, sleep disturbances, pain, and depression*.

* *Adjusted for age, sex, mRS score, sleep disturbances and pain*.

† *Defined as impairment in mental flexibility, fluences, working memory, or processing speed*.

**Figure 3:**
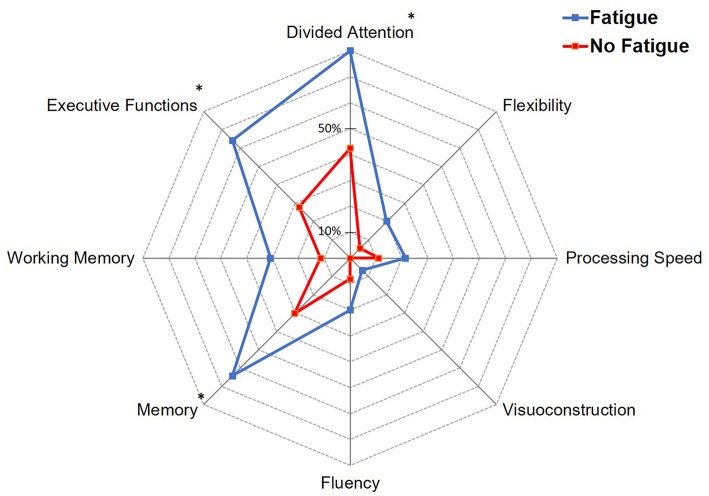
Proportion of non-depressed patients with impairment in the different cognitive domains according to fatigue. *p < 0.05.

Further analyses were restricted to the subgroup of non-depressed patients (*n* = 58, 84.1% of the total cohort). Among these patients, 41 (70.7%) had significant fatigue at 6 months. A higher proportion of patients with fatigue had a low score on MoCA scale (82.3 vs. 43.9% *p* = 0.07; OR = 5.96; 95% CI 1.48–24.0, *p* = 0.012). Figure 3 shows the proportion of non-depressed patients with abnormal scores in the different cognitive domains according to fatigue. Patients with fatigue had more frequently memory impairment (64.3 vs. 30.3%; OR = 4.14; 95% CI: 1.10–15.5, *p* = 0.035), executive dysfunction (64.3 vs. 27.8%; OR = 4.68; 95% CI: 1.26–17.4, *p* = 0.021), or deficit in divided attention task (80 vs. 42.4%; OR = 5.43; 95% CI: 1.00–29.6, *p* = 0.051). In multivariable logistic regression analysis, a score <26 on MoCA scale (OR 5.12; 95% CI: 1.00–26.2, *p* = 0.05) and a memory impairment (OR = 6.17; 95% CI: 1.06–35.9, *p* = 0.043) were associated with fatigue at 6 months in non-depressed patients. In addition, there was a non-significant trend toward an association between divided attention deficit and fatigue (OR = 6.79; 95% CI: 0.80–57.6, *p* = 0.079).

## Discussion

This study demonstrated that fatigue is a common symptom 6 months after ischemic stroke in patients who received acute revascularization therapy. Indeed, nearly a third of patients had significant fatigue. The frequency of fatigue observed in our population is nevertheless rather low when we consider the wide distribution of the prevalence of fatigue in epidemiological studies in which up to 77% of patients may suffer this symptom (4). Since fatigue is correlated with residual motor disability, the observed difference could be explained by the fact that our population was composed of patients with a low residual disability (72.9% of patients had a NIHSS = 0 score and 61.4% had a mRs score ≤ 1). A study conducted in patients with minor ischemic stroke (initial NIHSS score ≤ 6 and 6-month mRs score ≤ 1) found a prevalence of fatigue of 30%, which is consistent with our results (27). To account for the relationship between fatigue and residual handicap, it could be argue that physical activity is more important in patients with a good motor recovery thus improving fatigue through different mechanisms including aerobic training that could strengthen muscle capacity and allow better physical performance by reducing the feeling of fatigue. Physical exercise could also contribute to higher self-esteem and more social interaction (28).

Depression appears to be closely associated with fatigue. In our study 29% of patients with fatigue had depressive symptoms, which is consistent with previous studies (29). Fatigue has long been considered a symptom of post-stroke depression. Although fatigue and depression are closely related, it is now recognized that fatigue can exist independently of depression. Indeed, fatigue after ischemic stroke can occur in the absence of depression, which has also been highlighted in other neurological diseases including Parkinson's disease (30), and multiple sclerosis (31). The question remains open regarding the causal relationships between depression and fatigue: depression may in some cases be more a consequence than a causal factor of fatigue (32). Although patients with fatigue were older than those without fatigue, we did not find a significant statistical correlation between age and FSS score (Spearman coefficient: 0.11, *p* = 0.39). In the literature, the association between post-stroke fatigue and age has been inconsistently reported (4).

The presence of cognitive impairment appears to be associated with fatigue. Considering that depression may influence cognitive evaluations, we studied a subgroup of non-depressed patients. As a result, a low score on MoCA scale was associated with fatigue in non-depressed patients. Several studies did not report association between fatigue and cognitive impairment (33). This may be explained by the fact that these studies used the Mini-Mental State Examination (MMSE) score that have a lower sensitivity than the MoCA in assessing post-stroke cognitive impairment (34). Indeed, unlike the MMSE score, the MoCA scale allows an assessment of executive and attentional functions, which are frequently affected in vascular cognitive disorders.

When analyzing specifically cognitive domains, memory impairment was significantly associated with fatigue in both overall and non-depressed patients. One previous study found more frequent memory problems in patients with post-stroke fatigue compared with those without fatigue (35). Conversely, two studies did not conclude to such an association, but neuropsychological tests used for memory assessment differed from our study (27, 36). In addition, only minor strokes for one study and both minor strokes and transient ischemic attacks for the other were included. We also found a trend toward an association between fatigue and divided attention impairment in non-depressed patients, although statistical significance was not reached probably due to a lack of power. To the best of our knowledge, one study found an association between sustained attention deficit and fatigue 1 year after ischemic stroke, as well as with executive disorders in a group of non-depressed patients (27). Another study found attention deficit but in a more heterogeneous population that also involved patients with subarachnoid hemorrhage (37). Several studies have shown a correlation between impaired processing speed and fatigue after stroke (35–37). However, in one study, the deficit was only detected at 3 and did not persist after 6 months, the other two studies did not specify when the neuropsychological assessment was performed during follow-up.

Cognitive impairment has been shown to contribute to fatigue after subarachnoid hemorrhage (38) or traumatic brain injury (39). Our study would reinforce the idea that this may also be the case for ischemic stroke. If cognitive disorders and fatigue appear to be related, it is difficult to specify causal links. Ischemic stroke is an interesting condition for understanding major cognitive functions due to possible focal lesion. Some so-called strategic lesions have helped to establish neuroanatomical correlates of cognitive functions. Regarding fatigue, a pilot study conducted in patients with ischemic stroke who had no significant sequelae or mood disorders found that brainstem and thalamic lesions were more frequent in patients with fatigue (40). The authors suggested that so-called primary fatigue could be the result of minimal attention deficits secondary to an interruption of neural networks, particularly the reticular formation. This hypothesis was not confirmed by a subsequent study conducted in a larger number of patients, in which no association between fatigue and the side or site of the lesion was found (27). Since fatigue is a multidimensional and complex phenomenon, any injury can contribute to increasing the risk of fatigue due to its multiple expression.

Several limitations must be acknowledged. Despite a large screening, the number of patients finally included was limited, thus leading to a lack of power for some multivariable analyses. In a few cases, it was not possible to perform several neuropsychological tests, but there was no difference between patients with and those without fatigue. These limitations underline the fact that neuropsychological assessment after stroke, as well as evaluation of other functional outcomes including fatigue, is difficult and time consuming in routine clinical practice, which could explain the paucity of data in the literature. Finally, it was not possible to assess with the FSS which specific dimension of fatigue the patients complained with. Hence, it would be interesting to evaluate whether cognitive impairment could be more frequently observed in patients with mental fatigue rather than physical fatigue.

To conclude, fatigue after ischemic stroke is a frequent symptom even in patients treated with acute revascularization therapies and good functional recovery. Although it is difficult to establish a causal link, the association between fatigue and subtle cognitive impairment including memory or attention deficits could be of interest in developing further interventional studies so as to define therapeutic strategies to improve these symptoms.

## Data Availability

The datasets generated for this study are available on request to the corresponding author.

## Author Contributions

MGr: study concept and design, acquisition of data, neurological evaluation of patients, analysis and interpretation of data, and drafting and revising the manuscript for content. LG, GD, SM, BD, CB-La, JG, MH-B, G-VO, and MGi: acquisition of data, neurological evaluation of patients, and critical revision of manuscript for intellectual content. SG, OR, AP, CT, and CB-Le: acquisition of data, neuropsychological evaluation of patients, and critical revision of manuscript for intellectual content. YB: study concept and design, acquisition of data, neurological evaluation of patients, analysis and interpretation of data, study supervision, obtaining funding, drafting, and revising the manuscript for content.

### Conflict of Interest Statement

YB received honoraria for or consulting fees from AstraZeneca, Bayer, BMS, Pfizer, Medtronic, MSD, Amgen, and Boehringer-Ingelheim. The remaining authors declare that the research was conducted in the absence of any commercial or financial relationships that could be construed as a potential conflict of interest.
